# Effects of Short-Term Nitrogen Additions on Biomass and Soil Phytochemical Cycling in Alpine Grasslands of Tianshan, China

**DOI:** 10.3390/plants13081103

**Published:** 2024-04-15

**Authors:** Chao Liu, Junjie Liu, Juan Wang, Xiaoyu Ding

**Affiliations:** 1 College of Ecology and Environment, Xinjiang University, Urumqi 830017, China; 13353567402@163.com (C.L.); juanwang.33@foxmail.com (J.W.); dxy2078@163.com (X.D.); 2 Technology Innovation Center for Ecological Monitoring and Restoration of Desert-Oasis, Ministry of Natural Resources Desert, Urumqi 830002, China; 3 Key Laboratory of Oasis Ecology, Ministry of Education (Xinjiang University), Urumqi 830017, China

**Keywords:** nitrogen addition, alpine grassland, biomass, ecochemical characterization, structural equation modeling

## Abstract

The nitrogen deposition process, as an important phenomenon of global climate change and an important link in the nitrogen cycle, has had serious and far-reaching impacts on grassland ecosystems. This study aimed to investigate the survival adaptation strategies of plants of different functional groups under nitrogen deposition, and the study identified the following outcomes of differences in biomass changes by conducting in situ simulated nitrogen deposition experiments while integrating plant nutrient contents and soil physicochemical properties: (1) nitrogen addition enhanced the aboveground biomass of grassland communities, in which Poaceae were significantly affected by nitrogen addition. Additionally, nitrogen addition significantly influenced plant total nitrogen and total phosphorus; (2) nitrogen addition improved the plant growth environment, alleviated plant nitrogen limitation, and promoted plant phosphorus uptake; and (3) there was variability in the biomass responses of different functional groups to nitrogen addition. The level of nitrogen addition was the primary factor affecting differences in biomass changes, while nitrogen addition frequency was an important factor affecting changes in plant community structure.

## 1. Introduction

Grassland is one of the most important and widely distributed ecosystem types in terrestrial ecosystems [1,2], and they play an important role in the global carbon and nitrogen cycle, climate regulation, and in coping with global changes [3,4]. Grasslands in China cover an area of about 4.0 × 10^8^ hm^2^, accounting for about 41.7% of the total land area [5]. A significant portion of these grasslands are located in sensitive and critical zones of global climate change, making them important experimental sites for the study of nutrient cycling in grassland ecosystems in the context of global climate change.

Since the Industrial Revolution, the burning of fossil fuels and the extensive use of nitrogen fertilizers have caused a dramatic increase in nitrogenous compounds emitted into the atmosphere and deposited on the surface [6]. In China, nitrogen deposition saw a significant rise between 1980 and 2010, with an average growth rate of about 0.4 kg·N·ha^−1^·yr^−1^ [7], and in recent years, China has developed into the third-largest high-nitrogen-depositing area in the world after Europe and the United States [8]. As an important phenomenon of global climate change and an important link in the nitrogen cycle, the impact of the nitrogen deposition process on grassland ecosystems has become one of the research hotspots in ecology. Currently, research on nitrogen deposition in grassland ecosystems mainly focuses on the differences in plant productivity and changes in species diversity due to nitrogen addition [9,10,11]. In contrast, studies on physicochemical soil properties are less common. A close mutual feedback relationship exists between soil and plants during plant growth, with the soil providing the necessary nutrients for plant growth and development, and plants influencing the soil nutrients through, for example, apoplastic matter return [12,13]. The physicochemical soil properties tend to respond factors such as soil pH, total organic carbon, total nitrogen, and available phosphorus, tending to respond more directly and rapidly to nitrogen additions compared to the slow response of plant growth characteristics such as plant community and productivity. Tian et al. [14] found that nitrogen addition decreased the soil pH by a global meta-analysis, Xu et al. [15] found that nitrogen addition increased the soil total organic carbon by a global meta-analysis, Zhou et al. [16] found that nitrogen addition increased the soil nitrogen effectiveness by a global meta-analysis, and Lu et al. [17] found that nitrogen addition increased the soil available phosphorus content.

Moderate nitrogen input enhances ecosystem productivity by raising the soil nitrogen levels and stimulating microbial activity. This, in turn, promotes microbial nitrogen mineralization and boosts the soil’s nutrient content, making more nitrogen available for plant growth [18,19,20]. However, as nitrogen input continues to increase, humus decomposing enzymes produced by soil microorganisms decrease, microbial activity decreases, and soil nitrogen mineralization is weakened [21]. Once the ecosystem becomes saturated with nitrogen, the excess inorganic nitrogen undergoes nitrification and denitrification, which increases the risk of nitrogen loss [22,23]. Concurrently, increased nitrogen input boosts the accumulation of reactive nitrogen in the soil, which can lead to the rapid growth of nitrogen-loving plants within the community, thereby affecting the structure and function of the grassland ecosystem [24]. The impact of nitrogen deposition on ecosystems varies significantly based on climate zone, grassland system type, nitrogen addition level, nitrogen fertilizer type, and experiment duration [25,26], and some studies have shown that the frequency of nitrogen addition is also one of the important factors affecting the ecosystem effect [27]. Simultaneously integrating soil and plant stoichiometric characteristics in response to nitrogen addition and studying nutrient cycling from both plant–soil levels can more comprehensively explain the intrinsic and extrinsic factors of species diversity changes.

Bayanbulak Grassland, located in the Tianshan Mountains, is the largest subalpine alpine grassland in China and the second-largest alpine grassland in China [28]. According to the observation data of the Chinese Academy of Sciences, the environmental nitrogen deposition of the grassland is about 8 kg·N·ha^−1^·yr^−1^ [29], which is slightly lower than the normal critical load of global grassland nitrogen deposition (10–20 kg·N·ha^−1^·yr^−1^) [30], but given its unique geographical location and climatic conditions, it is particularly important to study the changes in the Bayanbulak alpine grassland ecosystem in the context of nitrogen deposition to predict future changes in grassland structure and function. In this study, we investigated the effects of increased nitrogen deposition on the physicochemical soil properties (soil water content, pH, soil salt content, total organic carbon, total nitrogen, and total phosphorus) and plant nutrient contents (total organic carbon, total nitrogen, and total phosphorus) of Bayanbulak alpine grassland through nitrogen addition experiments with different levels and frequencies, in order to provide a scientific foundation for the adaptive management of ecosystems against the background of increasing atmospheric nitrogen deposition. Additionally, our research aimed to address the following scientific inquiries: (1) Is there variability in the responses of grassland community biomass to different patterns of nitrogen addition? Is the frequency of nitrogen addition also an important factor causing changes in the plant community structure? (2) Is plant biomass growth directly affected by the physicochemical soil properties? Are there differences in the effects of the soil’s physical and chemical properties on plants of different functional groups? Are the influence pathways that cause changes in the biomass of different functional groups consistent?

## 2. Materials and Methods

### 2.1. Study Area

**Figure 1:**
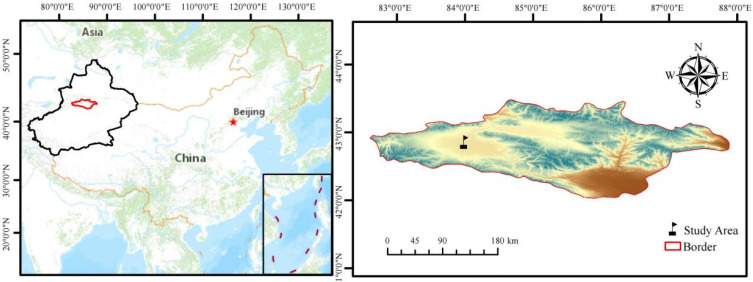
Study area.

The study area is located near the Bayanbulak Grassland Ecosystem Research Station of the Chinese Academy of Sciences (42°88′ N, 83°70′ E) in Hejing County, Bayin’guoleng Mongol Autonomous Prefecture, Xinjiang, China (Figure 1). This region has a typical alpine climate and an altitude of about 2470 m [31]. The average annual temperature is −4.6 °C, with an extreme maximum of 25.4 °C and an extreme minimum of −40.5 °C. The annual rainfall ranges from 216.8 to 316.8 mm, with an average annual rainfall of about 270 mm, of which 60% to 80% is concentrated in the growing season. The soil type of the study area is chestnut-calcium soil, which is flat and has a homogeneous distribution of grass species. The experimental treatments were preceded by five years of enclosure, and the vegetation type was alpine grassland dominated by perennial herbaceous plants, with the main species being *Festuca ovina* L. (Poaceae), *Agropyron cristatum* (Linn.) Gaertn. (Poaceae), *Potentilla fragarioides* L. (Rosaceae), *Potentilla bifurca* Linn. (Rosaceae), and *Astragalus adsurgens* (Fisch.) Bunge. (Fabaceae).

### 2.2. Experimental Design

**Figure 2:**
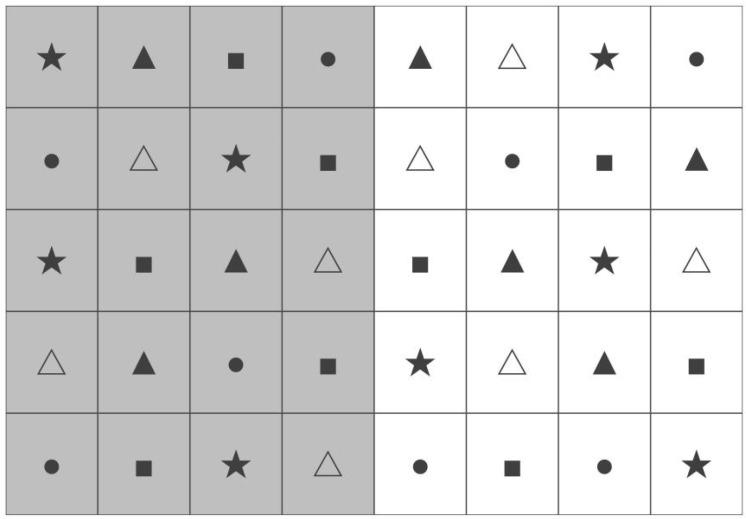
Sample distribution. Note: △: Control (CK) (0 g·m^−^^2^); ▲: Low N (LN) (5 g·m^−^^2^); ●: Medium N (MN) (10 g·m^−^^2^); ■: High N (HN) (15 g·m^−^^2^); and ★: Severe N (SN) (20 g·m^−^^2^); high-frequency nitrogen addition on the left and low-frequency nitrogen addition on the right.

The experiment was designed as a randomized block group experiment, and NH_4_NO_3_ (35%N) was selected as the sole nitrogen additive for the in situ simulated nitrogen addition experiments. A total of five nitrogen addition levels were set up in the experiments: Control (CK) (0 g·m^−^^2^), Low N (LN) (5 g·m^−^^2^), Medium N (MN) (10 g·m^−^^2^), High N (HN) (15 g·m^−^^2^), and Severe N (SN) (20 g·m^−^^2^), and two nitrogen addition frequencies: high-frequency nitrogen addition (3 lots of nitrogen addition) and low-frequency nitrogen addition (1 lot of nitrogen addition). Each treatment was set up with 4 replications. The experimental plots consisted of 40 3 m × 3 m plots with a 0.5 m buffer strip between plots (Figure 2). The low-frequency nitrogen addition was carried out at the end of April 2021, and the high-frequency nitrogen addition was divided into three groups at the end of April, May, and June 2021, respectively.

### 2.3. Sample Collection and Sample Determination Methods

#### 2.3.1. Sample Collection

Plant sample collection: At the end of August 2021, 40 0.5 m × 0.5 m sample squares were randomly set up within 40 sample squares, and all plants within the small sample squares were harvested flush and classified into archival bags according to species to be returned to the laboratory, placed in the oven to be killed first (105 °C, 30 min), and dried to a constant weight (65 °C, 48 h) to perform the weighing process (0.01 g).

Soil sample collection: After harvesting the sample plots, the surface withered material was removed in 40 small sample plots, and soil samples were collected by the soil auger method (d = 5 cm). In total, 3 soil augers were collected in each sample plot (classification according to 0–10 cm and 10–20 cm deep: 0–10 cm soil represents shallow soil and 10–20 cm soil represents deep soil) and mixed into 2 soil samples, totaling 80 soil samples. Each soil sample was divided into two parts, one part for soil water content determination and one part for soil physicochemical property determination, including pH, EC, soil TOC, soil TN, and soil TP.

#### 2.3.2. Sample Determination Methods

The dried plant samples were categorized based on different functional groups and then ground using a 100-mesh sieve to sieve to access the nutrient content. The air-dried soil samples were sieved through a 100-mesh sieve to determine the physical and chemical properties of the soil. The research methodology is as follows [32]:

The plant total organic carbon (Plant TOC) was determined by the K_2_Cr_2_O_7_-FeSO_4_ titration method. The soil sample was weighed using an electronic balance to weigh 0.03 g of a 100-mesh sieve of a plant sample (accurate to 0.0001 g) into a conical flask. Next, 10 mL of a 1/6 mol/L K_2_Cr_2_O_7_ solution (prepared by dissolving 49.04 g of K_2_Cr_2_O_7_ in solvent to a final volume of 1 L) was added to the flask, followed by thorough shaking to ensure proper mixing. Subsequently, 10 mL of 98% H_2_SO_4_ was added, and the mixture was shaken well and allowed to react for 30 min. After the reaction time had elapsed, 100 mL of distilled water was added to the flask to dilute the acidic mixture. The solution was then shaken well to ensure homogeneity. Following this, 5–8 drops of o-phenanthroline indicator solution (prepared by dissolving 1.485 g of o-phenanthroline and 0.795 g of FeSO_4_ in solvent to a final volume of 100 mL) were added, and the flask was shaken well once more to ensure an even distribution of the indicator. Finally, the solution was titrated with a 1/2 mol/L FeSO_4_ solution (prepared by dissolving 140 g of FeSO_4_ and 15 mL of 98% H_2_SO_4_ in solvent to a final volume of 1 L). The endpoint of the titration was indicated by a color change in the solution, which initially shifted from blue to green and then to red, signaling the completion of the reaction.

The plant total nitrogen (Plant TN) was determined by the semi-micro Kjeldahl method. A total of 0.05 g of the plant sample (accurate to 0.0001 g) was taken and placed at the bottom of a digestion tube. Then, 5 mL of 98% H_2_SO_4_ was added to the tube. Next, heating over a small flame in the digestion furnace was performed, waiting for the H_2_SO_4_ to produce white fumes. Then, the temperature was increased. Once the solution turned into a uniform brownish-black, it was removed from the heat and allowed to cool slightly. Ten drops of H_2_O_2_ were added and then the mixture was heated until it reached a gentle boil, cooking for about 7 to 10 min. After cooling slightly, the addition of H_2_O_2_ was repeated, followed by more heating. This process was repeated three times, with each subsequent addition of H_2_O_2_ being in smaller amounts. Cooking was continued until the solution was colorless or clear, and then it was heated for an additional 10 min to remove any remaining H_2_O_2_. After the solution cooled, it was transferred to a 100 mL volumetric flask using water, and filled to the mark once it reached room temperature. The digest was allowed to stand overnight. The next day, 5 mL of the clear solution was taken and 2 mL of 1/2 mol/L potassium sodium tartrate solution (prepared by dissolving 100 g in 1 L of water), 5 mL of 1.783 mol/L potassium hydroxide (prepared by dissolving 100 g in 1 L of water), 25 mL of distilled water, and 2.5 mL of Nasrid reagent (prepared by dissolving 45.0 g of HgI_2_ and 35.0 g of KI in 400 mL of distilled water, then transferring to a 1 L volumetric flask and adding 112 g of KOH. After adding distilled water up to 800 mL, it was mixed well, cooled to a volume of 1 L, left to stand overnight, and then filtered into a brown volumetric flask for later use) were added. Water was added to the mixture, shaken well to bring the volume up to 30 mL, and then a UV spectrophotometer was used to compare the color intensity at a wavelength of 425 nm. Finally, the mass of total nitrogen in the plant was determined using the standard curve.

The plant total phosphorus (Plant TP) was determined by the Mo-Sb Colorimetric method. In total, 0.05 g of the plant sample (accurate to 0.0001 g) was taken and placed at the bottom of a digestion tube. Next, 5 mL of 98% H_2_SO_4_ was added to the tube. Heating over a small flame in the digestion furnace was performed, waiting for the H_2_SO_4_ to produce white fumes. Then, the temperature was increased. Once the solution turned into a uniform brownish-black, it was removed from the heat and allowed to cool slightly. Ten drops of H_2_O_2_ were added and then the mixture was heated until it reached a gentle boil, cooking for about 7 to 10 min. After cooling slightly, the addition of H_2_O_2_ was repeated, followed by more heating. This process was repeated three times, with each subsequent addition of H_2_O_2_ being in smaller amounts. Cooking was continued until the solution was colorless or clear, then it was heated for an additional 10 min to remove any remaining H_2_O_2_. After the solution cooled, it was transferred to a 100 mL volumetric flask using water, and filled to the mark once it reached room temperature. The digest was allowed to stand overnight. A total of 5 mL of supernatant was taken after standing overnight, 2 drops of indicator were added, 10 mL of distilled water was added, the supernatant was adjusted to a light yellow color with 4 mol/L of NaOH (160 g NaOH was volume to 1 L), and then a drop of NaOH was added. NaOH (160 g NaOH to 1 L) adjusted it to a slight yellow, and then a few drops of 2 mol/L diluted H_2_SO_4_ (60 mL of concentrated H_2_SO_4_ to 1 L) adjusted this to fade. Molybdenum and antimony anti were added (first the molybdenum and antimony mixture was configured (A: 153 mL of concentrated H_2_SO_4_ added into 300 mL of distilled water, mixed and then cooled; 10 g of ammonium molybdate in the cooled H_2_SO_4_) and dissolved completely. B: Potassium antimony tartrate 0.5 g was fixed to a 100 mL volumetric flask. Solution B was poured into solution A to a 1 L brown volumetric flask), followed by 1.5 g of ascorbic acid into 100 mL of molybdenum and antimony mixture) at 5 mL, shaking well and then fixing to 50 mL. After 30 min, the absorbance was recorded by an ultraviolet spectrophotometer with a wavelength of 880 nm, and the total phosphorus content of the plant was calculated by the standard curve.

The soil pH was determined using a pH meter. To prepare the sample, a ratio of water to soil of 5:1 was used. Specifically, 10 g soil, which was sieved through a 20-mesh sieve, was weighed and placed into a triangular flask. Then, 50 mL of distilled water was added to the flask. The mixture was shaken on a shaker for 15 min to ensure thorough mixing and facilitate the release of ions from the soil particles into the water. After shaking, the flask was allowed to stand for 10 min to let the soil particles settle. Following this sedimentation period, the supernatant was carefully filtered through filter paper to obtain a clear solution. Finally, the pH of the clarified solution was measured using a calibrated pH meter. The reading was taken once the value on the pH meter stabilized, ensuring an accurate measurement of the soil pH.

The soil salt content (SSC) was determined using the potentiometric method. In this process, a soil-to-water ratio of 5:1 was used. The soil was mixed with water and shaken on a shaker for 15 min to ensure that the soluble salts were adequately dissolved. After shaking, the mixture was allowed to stand for 10 min, which allowed the soil particles to settle. The clear liquid, now known as the leachate, was then separated from the soil particles through filtration. The conductivity of the leachate was measured using a conductivity meter, which provided a quantitative assessment of the dissolved salt content in the soil. A plotted standard curve was used to convert the conductivity of the leachate and soil salinity.

The soil water content (SWC) was determined using the drying and weighing method. Initially, a dry aluminum box was taken and weighed, with this initial weight recorded as W_1_ (g). Subsequently, soil samples were carefully placed into the aluminum box, whose weight was predetermined, and the total weight was recorded as W_2_ (g). The box, now containing the soil, was then opened and placed in an oven, with the lid left ajar. The samples were dried at approximately 105 °C for 48 h until they reached a constant weight. After cooling, the box with the soil was weighed once more, and this final weight was recorded as W_3_ (g).

(1) $$\mathrm{SWC}\%=\frac{W_{2}-W_{3}}{W_{3}-W_{1}}\times 100\%$$

The soil total organic carbon (Soil TOC) was determined by the K_2_Cr_2_O_7_-FeSO_4_ titration method. The soil sample was weighed using an electronic balance to weigh 0.5 g of a 100-mesh sieve of soil sample (accurate to 0.0001 g) into a conical flask. Next, 10 mL of a 1/6 mol/L K_2_Cr_2_O_7_ solution (prepared by dissolving 49.04 g of K_2_Cr_2_O_7_ in solvent to a final volume of 1 L) was added to the flask, followed by thorough shaking to ensure proper mixing. Subsequently, 20 mL of 98% H_2_SO_4_ was added, and the mixture was shaken well and allowed to react for 30 min. After the reaction time had elapsed, 100 mL of distilled water was added to the flask to dilute the acidic mixture. The solution was then shaken well to ensure homogeneity. Following this, 5–8 drops of o-phenanthroline indicator solution (prepared by dissolving 1.485 g of o-phenanthroline and 0.795 g of FeSO_4_ in solvent to a final volume of 100 mL) were added, and the flask was shaken well once more to ensure an even distribution of the indicator. Finally, the solution was titrated with a 1/2 mol/L FeSO_4_ solution (prepared by dissolving 140 g of FeSO4 and 15 mL of 98% H_2_SO_4_ in solvent to a final volume of 1 L). The endpoint of the titration was indicated by a color change in the solution, which initially shifted from blue to green and then to red, signaling the completion of the reaction.

The soil total nitrogen (Soil TN) was determined by the semi-micro Kjeldahl method. The soil sample was weighed using an electronic balance (accurate to 0.0001 g) and placed into the bottom of the digestion tube, along with approximately 0.5 g of soil that was sieved through a 100-mesh sieve. Then, 2 g of the mixed accelerator (with a K_2_SO_4_:CuSO_4_:SeO ratio of 100:10:1) was added, followed by 5 mL of concentrated H_2_SO_4_. The mixture was then heated at 350 °C for 1.5 h, after shaking well, and then cooled and brought to a volume of 100 mL. After allowing the mixture to stand overnight, the supernatant was taken, and to it, 2 mL of KOH and a sodium tartrate solution (prepared by dissolving 100 g of sodium tartrate in enough volume to make 1 L) were added, along with another 2 mL of KOH. Subsequently, the supernatant from the soil sample, after it stood overnight, was added. Next, 2 mL of sodium tartrate solution (prepared by dissolving 100 g of sodium tartrate in enough volume to make 1 L), 5 mL of KOH solution (prepared by dissolving 100 g of KOH in enough volume to make 1 L), 25 mL of distilled water, and 25 mL of Ghana’s reagent was prepared by dissolving 45.0 g of HgI_2_ and 35.0 g of KI in 400 mL of distilled water, which was then transferred to a 1 L volumetric flask, and 112 g of KOH was added. After adding 800 mL of distilled water, the mixture was shaken well and cooled to a fixed volume. Subsequently, 2.5 mL of KOH solution was added to a 1 L volumetric flask, the mixture was shaken well, and the volume was adjusted to 50 mL. The absorbance was then measured using a UV spectrophotometer at a wavelength of 425 nm for 30 min. Finally, the mass of total nitrogen in the soil was determined using the standard curve. Subsequently, 2.5 mL of KOH solution was added to a 1 L volumetric flask, the mixture was shaken well, and the volume was adjusted to 50 mL. The absorbance was then measured using a UV spectrophotometer at a wavelength of 425 nm for 30 min. Finally, the mass of total nitrogen in the soil was determined using the standard curve.

The soil total phosphorus (Soil TP) was determined by the Mo-Sb Colorimetric method. The soil sample was weighed using an electronic balance (accurate to 0.0001 g), 0.5 g of soil through a 100-mesh sieve was weighed into the bottom of the decoction tube, 8 mL of concentrated H_2_SO_4_ and 10 drops of HClO_4_ were added, shaken well and decocted at 350 °C for 50 min, cooled, and then volumed to 100 mL. In total, 5 mL of supernatant was taken after standing overnight, 2 drops of indicator were added, 10 mL of distilled water was added, and the supernatant was adjusted to a light yellow color with 4 mol/L of NaOH (160 g NaOH was volume to 1 L), and then a drop of NaOH was added. NaOH (160 g NaOH to 1 L) adjusted this to a slight yellow, and then a few drops of 2 mol/L dilute H_2_SO_4_ (60 mL of concentrated H_2_SO_4_ to 1 L) adjusted it to fade. Molybdenum and antimony anti were added (first, the molybdenum and antimony mixture was configured (A: 153 mL of concentrated H_2_SO_4_ was added into 300 mL of distilled water, mixed and then cooled; 10 g of ammonium molybdate in the cooled H_2_SO_4_) and dissolved completely. B: Potassium antimony tartrate 0.5 g was fixed to a 100 mL volumetric flask. Solution B was poured into solution A to 1 L brown volumetric flask), followed by 1.5 g of ascorbic acid into 100 mL molybdenum and antimony mixture) at 5 mL, shaken well and then fixed to 50 mL. After 30 min, the absorbance was recorded by an ultraviolet spectrophotometer with a wavelength of 880 nm, and the total phosphorus content of the soil was calculated by the standard curve.

### 2.4. Data Handling

Excel 2010 was used to summarize and organize the data, SPSS 24.0 was used to statistically analyze the data, and R 4.2.3 was used to plot the graphs. One-way analysis of variance (One way-ANOVA) was used to analyze the effects of nitrogen addition on the plant aboveground biomass, plant nutrient content, and physicochemical soil properties. A redundancy analysis was used to explore the significant silvers affecting biomass changes, and structural equation modeling (SEM) was used to explore the pathways of the aboveground biomass in response to nitrogen addition. The program packages ggplot2, ggcor, ggpubr, and piecewiseSEM were mainly used in this study.

## 3. Results

### 3.1. Plant Aboveground Biomass Response to Nitrogen Addition

As indicated in Table 1, the aboveground biomass of the grassland communities significantly increased (*p* < 0.05) with the increase in nitrogen fertilizer application level. Under the low-frequency nitrogen addition treatments, LN, MN, HN, and SN, the aboveground biomass increased by 4.98%, 10.68%, 35.03%, and 36.80%, respectively, compared to the CK treatment. Conversely, under the high-frequency nitrogen addition treatments, LN, MN, HN, and SN, the increases in aboveground biomass were −1.81%, 39.27%, 32.01%, and 51.77%, respectively, compared to the CK treatment. The aboveground biomass of the grassland, under both nitrogen addition modes, was greatly influenced by the level of nitrogen addition and was less influenced by the frequency of nitrogen addition.

**Figure 3:**
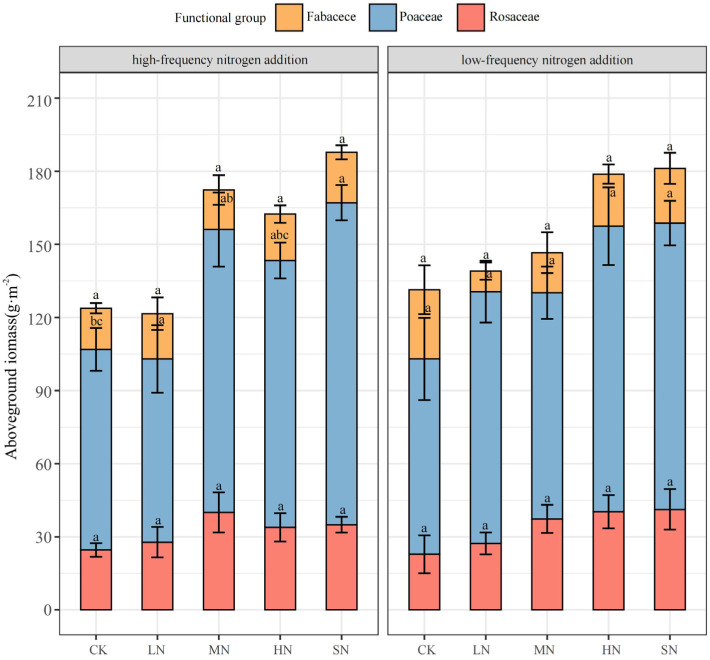
Effects of nitrogen addition on aboveground biomass of different functional groups. Note: Mean ± standard error. Different lowercase letters indicate significant differences between treatments (*p* < 0.05).

The aboveground biomass of the grassland communities significantly increased under both nitrogen addition modes. To explore the differences in nutrient utilization efficiency among plants of different functional groups within these grassland communities, we analyzed the changes in aboveground biomass based on functional group classification. The aboveground biomass of Poaceae was notably higher than that of Rosaceae and Fabaceae. Additionally, there were considerable variations in the response patterns of plants from different functional groups to nitrogen addition, as depicted in Figure 3. Specifically, the changes in the aboveground biomass of Poaceae plants were significantly (*p* < 0.05) influenced by nitrogen addition, with low-frequency nitrogen addition leading to increases of 28.94%, 15.81%, 46.19%, and 46.54%, respectively, and high-frequency nitrogen addition resulting in increases of −8.62%, 41.03%, 33.03%, and 60.53%, respectively, compared to the CK treatment. In contrast, the changes in the aboveground biomass of Rosaceae and Fabaceae were less influenced by nitrogen addition (*p* > 0.05). Low-frequency nitrogen addition caused increases of 19.35%, 63.49%, 76.40%, and 80.78% in Rosaceae, and decreases of 70.28%, 42.18%, 24.75%, and 20.99% in Fabaceae. High-frequency nitrogen addition improved the aboveground biomass of Rosaceae by 12.93%, 62.71%, 37.82%, and 42.25%, and Fabaceae by 9.88%, −3.96%, 12.71%, and 22.35%, respectively. As the absolute dominant species, Poaceae exhibited a significantly higher nitrogen utilization efficiency compared to Rosaceae and Fabaceae. Notably, the trend of change for Fabaceae differed between the two nitrogen addition modes, with legume biomass decreasing under low-frequency nitrogen addition and increasing under high-frequency nitrogen addition.

### 3.2. Response of Plant Chemical Traits to Nitrogen Addition

**Figure 4:**
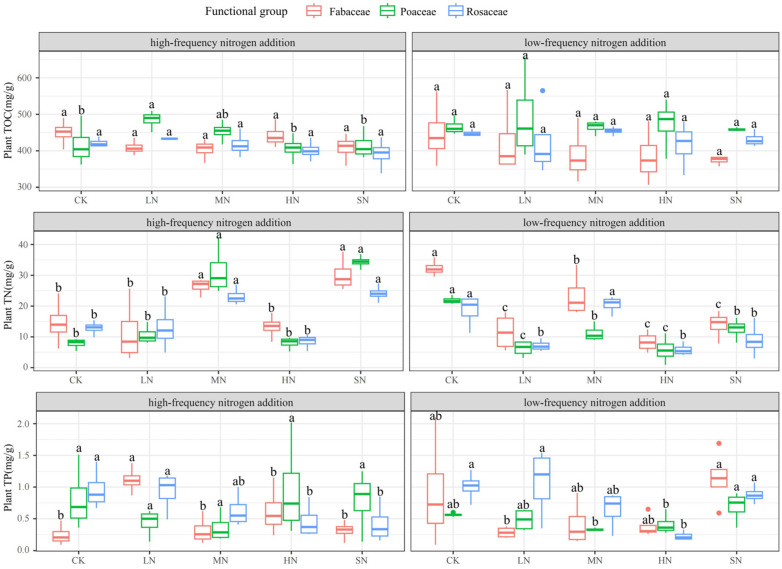
Effects of nitrogen addition on TOC, TN, and TP of plants of different functional groups. Note: Different lowercase letters indicate significant differences between treatments (*p* < 0.05).

Nitrogen addition had less effect on the plant TOC and more effect on the plant TN and TP, as shown in Figure 4. Under low-frequency nitrogen addition, the TOC of Poaceae was significantly higher than that of Rosaceae and Fabaceae, and the pattern of change in the TOC among plants of different functional groups was consistent. With high-frequency nitrogen addition, only the TOC of Poaceae showed a significant difference (*p* < 0.05), and the change patterns for the TOC in Poaceae and Rosaceae were similar, while the change in Fabaceae was more distinct. The changes in the TN and TP across the three functional groups were consistent under both nitrogen addition modes, and there were significant differences in the plant TN and TP at various nitrogen addition levels. Low-frequency nitrogen addition significantly influenced the TN and TP in all three functional groups. High-frequency nitrogen addition, on the other hand, had a significant impact on the TOC, TN, and TP of Poaceae, as well as the TN and TP of Rosaceae and Fabaceae.

**Figure 5:**
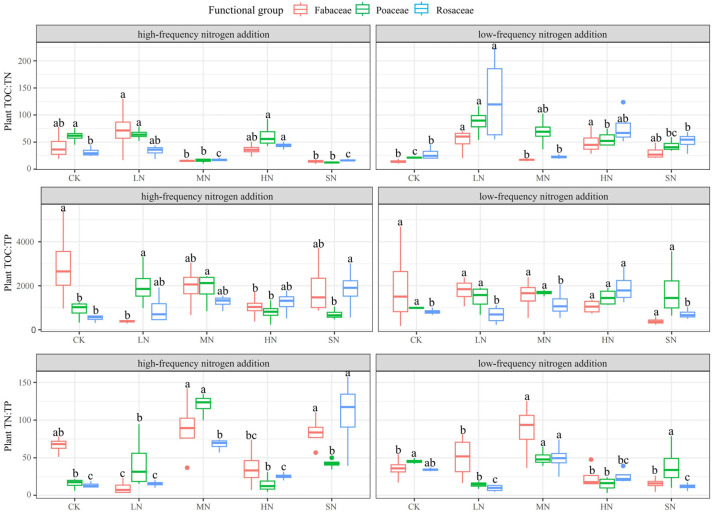
Effects of nitrogen addition on TOC:TN, TOC:TP, and TN:TP of different functional groups. Note: Different lowercase letters indicate significant differences between treatments (*p* < 0.05).

Nitrogen addition had a substantial impact on the ratios of plant TOC:TN, TOC:TP, and TN:TP, as illustrated in Figure 5. The patterns of change for TOC:TN and TN:TP among the three functional groups of plants were consistent under both nitrogen addition modes. However, differences among the functional groups were observed, suggesting that, while these plants shared a similar pattern in nitrogen uptake and utilization, they exhibited varying sensitivities to the added nutrients. The TOC:TN trends among the three functional groups were consistent under both addition modes, with higher TOC:TN values observed under the LN and HN treatments. The TOC:TP trends varied significantly among the groups: under low-frequency addition, Poaceae had a higher TOC:TP in CK, Rosaceae in HN, and Fabaceae in SN. With high-frequency addition, Poaceae showed a higher TOC:TP in CK, MN, and SN, while Rosaceae exhibited a higher TOC:TP under SN, and Fabaceae had a higher TOC:TP in LN and MN. The TN:TP trends were largely similar for the three functional groups under both addition modes, with MN treatments resulting in a higher TN:TP under low-frequency addition and both MN and HN treatments under high-frequency addition. Low-frequency nitrogen addition significantly influenced TOC:TN and TN:TP in Poaceae and Fabaceae, as well as TOC:TN, TOC:TP, and TN:TP in Rosaceae. High-frequency nitrogen addition had a significant impact on TOC:TN, TOC:TP, and TN:TP across all three functional groups of plants.

### 3.3. Response of Physicochemical Soil Properties to Nitrogen Addition

**Figure 6:**
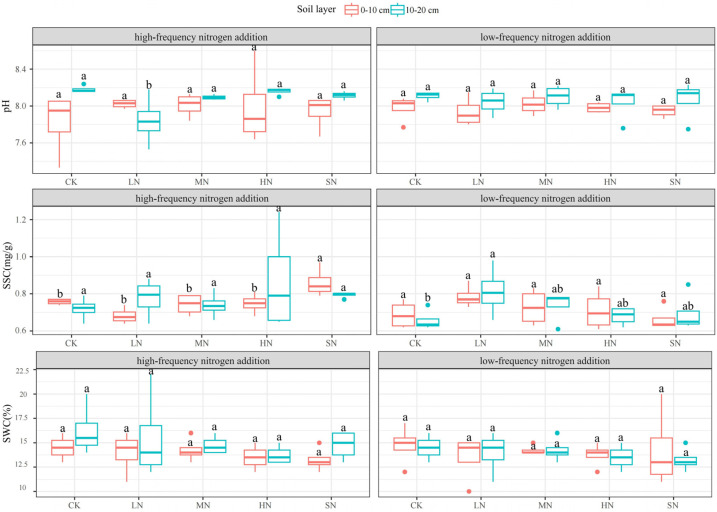
Effect of nitrogen addition on pH, SSC, and SWC in different soil layers. Note: Different lowercase letters indicate significant differences between treatments (*p* < 0.05).

Nitrogen addition had less effect on SWC and a greater effect on the soil pH and SSC, as shown in Figure 6. The 10–20 cm soil layer pH was significantly higher than that of the 0–10 cm soil layer under both nitrogen addition modes, there was no significant difference (*p* > 0.05) in the 0–10 cm and 10–20 cm soil layer pHs under low-frequency nitrogen addition, and there was a significant difference (*p* < 0.05) in the 10–20 cm soil layer pH under high-frequency nitrogen addition, with LN being significantly lower than that of the other treatments. SSC varied significantly under both nitrogen addition modes, with a significant difference in SSC from the 10–20 cm soil layer under low-frequency nitrogen addition, reaching a maximum value of 0.81 mg/g in LN, and a significant difference in SSC from the 0–10 cm soil layer under high-frequency nitrogen addition, reaching a maximum value of 0.86 mg/g in SN SSC. There was no significant difference in the soil SWC between the two nitrogen addition modes, where the 10–20 cm soil layer SWC was higher than the 0–10 cm soil layer SWC under high-frequency nitrogen addition. Low-frequency nitrogen addition significantly affected the 10–20 cm soil layer SSC, while high-frequency nitrogen addition significantly affected the 0–10 cm soil layer SSC and 10–20 cm soil layer pH.

**Figure 7:**
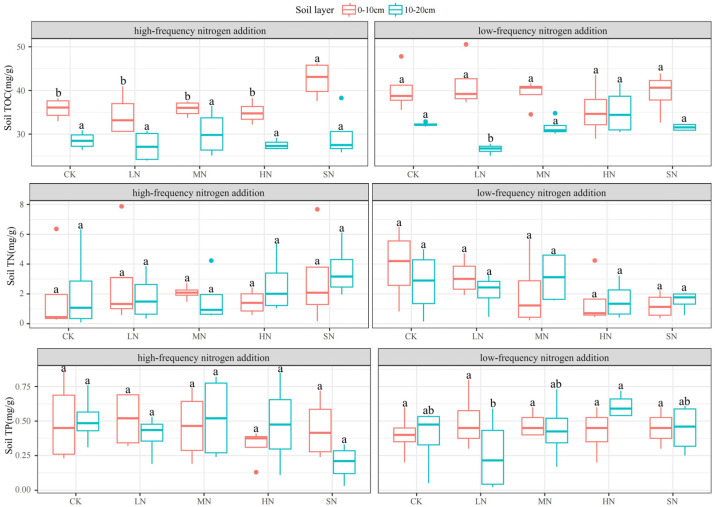
Effect of nitrogen addition on TOC, TN, and TP in different soil layers. Note: Different lowercase letters indicate significant differences between treatments (*p* < 0.05).

Nitrogen addition had less effect on the soil TN and a greater effect on the soil TOC and TP, as illustrated in Figure 7. The 0–10 cm soil layer TOC was significantly higher than the 10–20 cm soil layer TOC under both nitrogen addition modes, with the low-frequency nitrogen addition significantly affecting the 10–20 cm soil layer TOC (*p* < 0.05) and the high-frequency nitrogen addition significantly affecting the 0–10 cm soil layer TOC. There was no significant difference in the soil TN between the two soil layers under both nitrogen addition modes (*p* > 0.05). Low-frequency nitrogen addition significantly affected the 10–20 cm soil layer TP, and high-frequency nitrogen addition did not significantly affect the soil TP in either soil layer.

**Figure 8:**
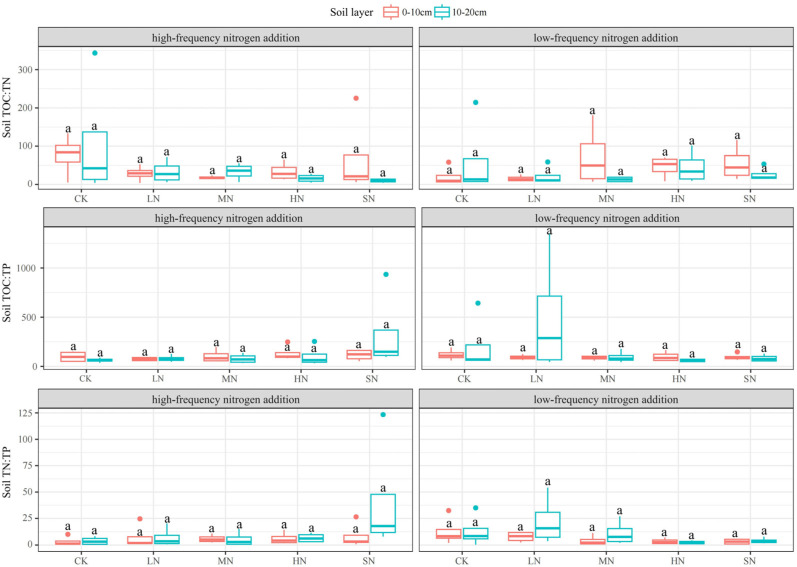
Effect of nitrogen addition on TOC:TN, TOC:TP, and TN:TP in different soil layers. Note: Different lowercase letters indicate significant differences between treatments (*p* < 0.05).

The effects of nitrogen addition on the soil TOC:TN, TOC:TP, and TN:TP were small, as shown in Figure 8. There were large differences in the patterns of change in the three indexes between different soil layers under the two nitrogen addition modes, but the similarity of the patterns of change in the TOC:TP and TN:TP in the same soil layer under the same nitrogen addition mode was high (TOC:TP and TN:TP in the 0–10 cm soil layer showed a decreasing trend with low-frequency nitrogen addition, and increased and then decreased in the 10–20 cm soil layer; TOC:TP and TN:TP in the 0–10 cm soil layer showed an increasing trend with high-frequency nitrogen addition, and increased and then decreased in the 10–20 cm soil layer). There were no significant differences in the TOC:TN, TOC:TP, and TN:TP between the 0–10 cm and 10–20 cm soil layers under the two nitrogen addition modes (*p* > 0.05).

### 3.4. Redundant Analysis of Biomass Changes

**Figure 9:**
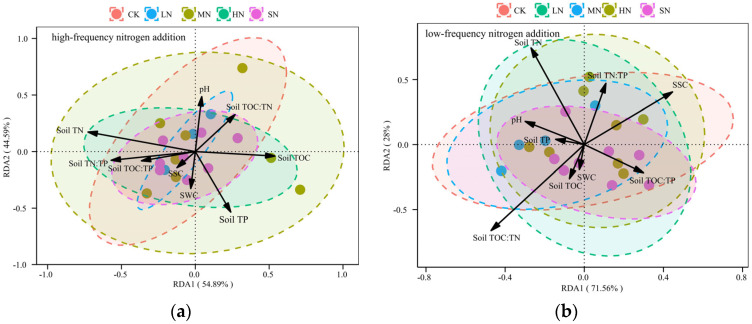
(**a**) RDA analysis of changes in biomass with high-frequency nitrogen addition and (**b**) RDA analysis of changes in biomass with low-frequency nitrogen addition. Note: The RDA result plot utilizes points to represent biomass samples, while arrows emanating from the origin symbolize different environmental factors. The length of these arrows signifies the strength of the influence that each environmental factor has on the changes in the aboveground community, with longer arrows indicating a stronger influence. The angle between the arrows and the coordinate axis represents the degree of correlation between the environmental factor and the axis, with smaller angles indicating a higher correlation. The vertical distance from a sample point to the extension line of an environmental factor’s arrow indicates the strength of the factor’s influence on that sample; the closer the sample point is to the arrow, the more significant the effect of the environmental factor on the sample. Furthermore, if a sample is situated in the direction of an arrow, it suggests a positive correlation between the environmental factor and the changes in the sample’s species community. Conversely, if a sample is located in the opposite direction of the arrow, it implies a negative correlation with the changes in the sample’s species community.

There was a strong correlation between plant biomass and soil environmental factors in both modes of nitrogen addition, as shown in Figure 9. In the redundancy analysis (RDA) constructed with low-frequency nitrogen addition, the cumulative explanation rate of the soil environmental factors on plant biomass was 99.56%, with Axis 1 accounting for 71.56% and Axis 2 accounting for 28.00%. In the RDA constructed with high-frequency nitrogen addition, the cumulative explanation rate of the soil environmental factors on plant biomass was 99.48%, with Axis 1 explaining 54.89% and Axis 2 explaining 44.59%.

### 3.5. Impact Pathway Analysis of Biomass Changes in Different Functional Groups

**Figure 10:**
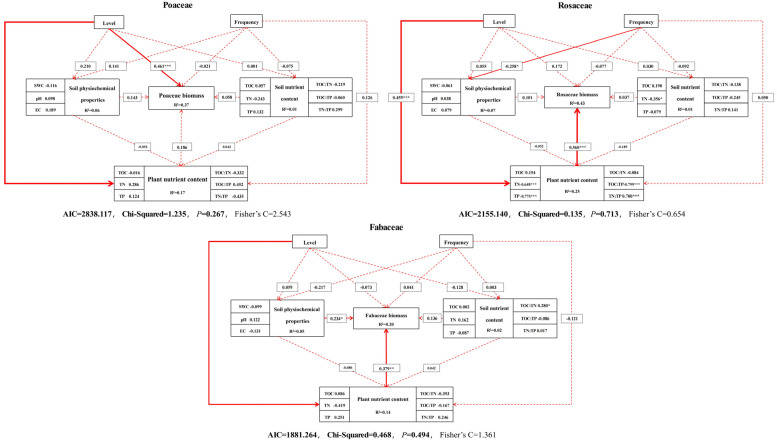
Pathway analysis of the effects of biomass change. Note: Continuous and non-continuous arrows represent direct and indirect relationships between variables, respectively. The width of the arrows corresponds to the significance of the path coefficients, with wider arrows indicating more significant relationships at the following levels: *** for *p* < 0.001, ** for *p* < 0.01, and * for *p* < 0.05, indicating a statistically significant direct or indirect effect.

The modeling process of structural equation models is driven by theoretical assumptions and is suitable for verifying and exploring complex direct and indirect causal relationships between multiple variables. In order to explore the differences in the responses of plant biomass to nitrogen addition in different functional groups, as well as the relationship between soil and plant chemical cycles, we selected the level and frequency of nitrogen addition as the independent variables of the model, selected physiochemical soil properties, soil nutrient content, and plant nutrient content as the observed variables of the model, and biomass as the dependent variable of the model, and constructed a complete model with all possible pathways. The SEM reflected the direct or indirect relationship between the nitrogen addition level and frequency on the differences in the effects of plant biomass of different functional groups (Figure 10). The SEM of Poaceae constructs explained 0.06, 0.01, 0.17, and 0.37 for physiochemical soil properties, soil nutrient content, plant nutrient content, and biomass, respectively, and the nitrogen addition level directly affected the Poaceae nutrient content and biomass. The SEM of Rosaceae constructs explained 0.07, 0.01, 0.25, and 0.43 for physiochemical soil properties, soil nutrient content, plant nutrient content, and biomass, respectively. Nitrogen addition level directly affected the Rosaceae nutrient content, nitrogen addition frequency directly affected the physiochemical soil properties, and plant nutrient content directly affected the Rosaceae biomass. The SEM of Fabaceae constructs explained 0.05, 0.02, 0.14, and 0.20 for physiochemical soil properties, soil nutrient content, plant nutrient content, and biomass, respectively, and nitrogen addition level directly affected the Fabaceae nutrient content, and the plant nutrient content and physiochemical soil properties directly affected the Fabaceae biomass.

## 4. Discussion

### 4.1. Nitrogen Addition Promotes Aboveground Biomass Growth

The biomass dynamics of grassland communities are a centralized expression of community structure and function, which can reflect the growth status of vegetation and its response to external environmental changes [33]. The results of simulated nitrogen deposition experiments conducted globally have shown that moderate nitrogen addition promotes the growth of the aboveground biomass of plants [34,35], while excessive nitrogen addition affects the diversity of grassland communities [36,37]. Our results showed a significant increase in aboveground biomass in both nitrogen addition modes, reaching a maximum at SN (181.17 g·m^−2^ for low-frequency and 187.88 g·m^−2^ for high-frequency nitrogen addition), which indicated that plant growth in this ecosystem was severely limited by nitrogen, and that a nitrogen addition of 20 g·m^−2^·a^−1^ did not reach the threshold for nitrogen limitation in this ecosystem. The results of the aboveground biomass survey based on functional groups showed that Poaceae biomass was significantly higher than that of Rosaceae and Fabaceae, and the aboveground biomass of Poaceae was more significantly affected by nitrogen additions, which is consistent with the findings of Van Sundert et al. [38] and He et al. [39]. The addition of exogenous nutrients has shifted plant competition from competition for limiting elements such as nitrogen and phosphorus to competition for light [40]. Under light competition conditions, an increase in nutrients leads to a decrease in light transmission in the lower layers of the plant and changes in habitat conditions for some species in the community, producing competitive exclusion [41]. Based on their advantage in morphology, Poaceae dominate rapid growth in light competition, increasing the effect of light stress on Rosaceae and Fabaceae, so the aboveground biomass of Poaceae plants is more significantly affected by nitrogen addition. There were differences in the change trends of Fabaceae under the two nitrogen addition modes, in which low-frequency nitrogen addition inhibited the growth of Fabaceae, while high-frequency nitrogen addition promoted the growth of Fabaceae, which was related to the nitrogen fixation ability of Fabaceae, which makes them able to biologically fix nitrogen by mutualistic symbiosis with rhizobacteria and be less dependent on effective soil nitrogen [42].

### 4.2. Nitrogen Addition Exacerbates P Limitation of Plant Growth

Plant nutrient content reflects the ability of the plant root system to obtain each nutrient element from the soil, as well as the migration and distribution relationship within the plant body, and the adequacy of nutrient content is an important indicator of plant growth conditions [43]. Both deficiencies and excesses of nutrients can have serious negative effects on plant growth, and maintaining relatively stable nutrient ratios in plant tissues is essential for healthy plant growth [44]. Plant nutrient content can reflect the survival strategy of plants, their growth rate, and their efficiency of nitrogen and phosphorus utilization, and the plant TOC:TN and TOC:TP reflect the growth rate of plants (TOC:TN responds to the efficiency of nitrogen utilization by plants and TOC:TP responds to the efficiency of phosphorus utilization by plants) and the TN:TP reflects the growth-restricted status of plants [45,46]. In the process of long-term coordinated evolution between plants and their environment, the structural substances (C) and non-structural species (N and P) composing the plant body will show differences in their responses to environmental changes, in which the plant TOC is less fluctuated by the environment and the TN and TP are more changed by the environment [44], which is consistent with our findings. The small changes in the TOC of the three functional groups of plants under exogenous nitrogen addition were due to the fact that carbon is the most basic element that constitutes the organism. Moderate nitrogen additions can promote plant growth and enhance photosynthesis and carbon fixation in plants. However, there is a threshold for plant nitrogen demand, beyond which, plant growth and carbon fixation may not increase significantly, thus limiting the effect of nitrogen addition on plant TOC. Thus, the effect of nitrogen addition on the TOC of the plants was small and did not show statistically significant differences. Nitrogen and phosphorus are the important elements that constitute the organism, and they are also the main factors of plant growth limitation. The significant changes in the TN and TP of the plants of the different functional groups under nitrogen addition indicated that short-term nitrogen addition alleviated plant nitrogen limitation and favored plant leaf nitrogen acquisition, so nitrogen additions would also promote plant phosphorus uptake by changing the effective phosphorus content in the soil [47]. Through the analysis of phytochemical characteristics, it was found that the TOC:TN and TN:TP variation rules of different functional groups of plants were consistent, while the variation rules of TOC:TP varied greatly, which indicated that the three functional groups of plants were basically consistent with the nitrogen utilization rules, and there were large differences in the efficiency of phosphorus utilization. Koerselman and Meuleman [48] suggested that plant growth is nitrogen-limited for plants with N:P < 14, P-limited for plants with N:P > 16, and potentially co-limited by both nitrogen and phosphorus for 14 < N:P < 16. Gusewell [49] suggested that N:P < 10 and N:P > 10 are more appropriate for determining nitrogen and phosphorus nutrient limitation types. Our results indicated that the plant growth in the study area was co-limited by nitrogen and phosphorus (9.70:1–87.31:1 for low-frequency nitrogen addition TN:TP and 10.38:1–120.55:1 for high-frequency nitrogen addition TN:TP), but that plant demand for phosphorus was higher as the level of nitrogen addition increased.

### 4.3. Nitrogen Addition Improves Plant Growth

Physiochemical soil properties are important indicators of soil quality, in which SWC promotes crop growth and increases soil nutrient content [50,51], and soil pH and SSC affect plant physiological responses, and them being too high or too low interferes with the efficiency of plant nutrient uptake [52]. Studies have shown that nitrogen addition decreases soil pH and increases SSC [53,54], while having less effect on SWC [55], which is not entirely consistent with our findings. The results of our study showed that there was no significant difference in SWC and an increase in SSC between the two nitrogen addition modes. The small difference in SWC may have been due to the increase in soil water use efficiency by the plants after nitrogen addition, while the soil evaporation capacity was reduced by the shading effect of plants, which balanced each other; the increase in SSC may have been due to the incomplete use of applied nitrogen fertilizer, which was enriched in the soil. Our study found no significant effect of nitrogen addition on the soil pH, which differs from previous studies, where numerous fertilization trials have shown that the application of nitrogen fertilizers leads to the accumulation of NO_3_^−^ and NH_4_^+^ in the soil, and that the uptake of NH_4_^+^ by a plant releases H^+^ into the soil solution, leading to a decrease in the soil pH [56]. In our study, we found significant differences in the soil pH only in the 10–20 cm soil layer under high-frequency nitrogen additions, while there were no significant differences in any of the other soil layers, which may have been caused by the short duration of fertilizer application, and the high concentration of nitrogen additions did not lead to soil acidification.

The soil nutrient content determines the state of plant growth and development and reflects the ability of the soil to provide nutrients to plants and improve environmental conditions [57]. Soil organic carbon is an important component of the soil and plays an important role in soil function and ecosystem value, and nitrogen addition can inhibit the microbial decomposition of plant-sourced organic matter inputs, as well as attenuate mineral adsorption by dead microbial residues, which can lead to changes in the carbon fraction [58]. Xu et al. [15] found that nitrogen addition significantly increased global organic carbon by 4.2%, and nitrogen addition increased the plant carbon input to the soil and reduced carbon loss during microbial decomposition and amplification, which is consistent with our findings. Nitrogen is an important indicator of soil fertility and plays an important role in plant growth [59], and some studies have shown that increased nitrogen deposition can increase the soil nitrogen content, with most of the nitrogen input to the soil being retained in soil organic matter and its mineralization playing a key role in soil fertility and plant nutrition [60], while our study showed that nitrogen addition had a small effect on the soil TN, which may have been related to the short duration of fertilizer application. After nitrogen application, the soil TN was maintained at a constant level due to the volatilization of most of the urea in the form of ammonia, coupled with the plant and microbial utilization of inorganic nitrogen and leaching of nitrate nitrogen. Nitrogen addition can cause soil acidification to increase the diffusion of phosphate ions and phosphorus availability and effectiveness, and it can also promote or inhibit the activity of soil phosphatase to increase or decrease the mineralization of soil organic phosphorus [61,62]. The results of our study showed that only with low-frequency nitrogen additions did the 10–20 cm soil layer TP change significantly, which may have been related to the effect of nitrogen addition in promoting the uptake of phosphorus by plants [63]. Soil C:N:P is an important parameter characterizing the soil C, N, and P balance, and plays an important role in judging the soil element limitation status and element geochemical cycle release. Huang et al. [64] showed that short-term nitrogen additions had a small effect on soil C:N:P in desert grasslands, and our results also showed that there were no significant differences in the soil TOC:TN, TOC:TP, and TN:TP between the two nitrogen addition modes. Nutrient cycling in soils is intertransformed between microorganisms, plants, and animals, and these complex interactions buffer the effects of nitrogen addition on the soil C:N:P. Furthermore, through the coupling of different soil chemical elements, nitrogen addition may enhance the cycling of certain nutrients, such as N. Meanwhile, other soil elements, like elemental C and P, interact with N through their respective cycling processes, thus contributing to the maintenance of the relative stability of soil C:N:P ratios. Meanwhile, we also found that the patterns of TOC:TP and TN:TP in the same soil layer were highly similar under the same nitrogen addition modes. In contrast, significant differences were observed between different nitrogen addition modes. This indicates that the frequency of nitrogen addition is an important factor affecting the physicochemical soil properties.

### 4.4. Nitrogen Addition Level Key to Plant Productivity

The RDA analysis revealed that plant biomass was strongly correlated with soil environmental factors under both nitrogen addition modes. However, the correlation between soil environmental factors and grassland productivity varied under different nitrogen addition modes. This suggests that the frequency of nitrogen addition is a significant factor contributing to differences in community structure. Furthermore, the frequency of nitrogen application may induce changes in the structure and function of soil microbial communities. Consequently, this can impact the soil nutrient cycling and plant nutrient uptake. N and P are the main limiting elements for plant growth in terrestrial ecosystems, and many nutrient addition experiments have shown that the diversity of grassland plants decreases significantly with nutrient addition, which is fundamentally due to the variability in the responses of different functional groups of plants to nutrient addition. The responses of grassland communities under natural or anthropogenic disturbances are usually complex and variable, and functional group-based studies can comprehensively evaluate the responses of functional groups and communities to environmental fluctuations [65]. In resource-poor habitats, different functional groups adopt different strategies in response to limiting factors, thereby influencing the competitive hierarchy that determines community composition and structure [66]. In order to reveal the response mechanisms of plants of different functional groups to nitrogen addition, we constructed SEM to analyze the influence pathways of biomass changes. The results showed that the nitrogen addition level had a direct effect on the aboveground biomass of Poaceae and an indirect effect on the aboveground biomass of Rosaceae and Fabaceae, which was consistent with the previous findings, and we speculated that it might be related to the nutrient utilization efficiency of plants. Nitrogen addition levels directly affect plant nutrient content and, thus, plant biomass, but the extent of this effect varies considerably, with indirect effects on biomass in Poaceae and direct effects in Rosaceae and Fabaceae, which also validates our conjecture above. Fayiah et al. [67] argued that physicochemical soil properties are important factors influencing changes in plant communities, and our results similarly prove the argument that physical soil properties directly affect Fabaceae biomass, and soil TN was significantly related to Rosaceae biomass. In addition, the frequency of nitrogen addition was also an important factor influencing the changes in plant community structure, and this argument can be verified from the significance of its influence pathway on plant biomass and the path coefficient.

## 5. Conclusions

The purpose of this paper was to explore the response characteristics of the physicochemical soil properties and phytochemical characteristics of Bayanbulak grassland to nitrogen deposition, as well as the influence pathways of the differences in the biomass changes of different functional groups. The results of this study show that: (1) The aboveground biomass of the two nitrogen addition modes increased significantly and reached the maximum value in SN, the growth of the plants in the study area was restricted by the joint limitation of nitrogen and phosphorus, and the addition of nitrogen exacerbated the limitation of the phosphorus element on the growth of the plants; (2) physicochemical soil properties will directly affect plant growth, there is variability in the responses of biomass to nitrogen addition in different functional groups, the level of nitrogen addition is a major factor affecting the differences in changes in biomass, and the frequency of nitrogen addition is also an important factor affecting the changes in plant community structure.

Carrying out research on the response mechanisms of Bayanbulak grassland under different nitrogen addition modes can help us to understand the potential change trends of the physiological and ecological characteristics of grassland against the background of increasing atmospheric nitrogen deposition, which is of great theoretical and practical significance for the study of the future responses and adaptations of grassland ecosystems in China to increasing atmospheric nitrogen deposition, and can also provide a theoretical basis and data support for the study of the carbon balance of the region and the sustainable development of grassland.

## Figures and Tables

**Table 1:** Effect of nitrogen addition on aboveground biomass of grassland community.

AGB (g·m^−2^)	Nitrogen Addition Levels				
	CK	LN	MN	HN	SN
High-frequency nitrogen addition	123.79 ± 8.13 b	121.55 ± 9.94 b	172.40 ± 16.14 a	163.42 ± 4.36 a	187.88 ± 3.48 a
Low-frequency nitrogen addition	132.43 ± 9.77 b	139.03 ± 12.62 b	146.57 ± 4.18 ab	178.82 ± 18.86 a	181.17 ± 8.11 a

Note: Mean ± standard error. Different lowercase letters indicate significant differences between treatments (*p* < 0.05).

## Data Availability

The data presented in this study are available on request from the corresponding author.

## References

[1] Zhu X., Zheng J., An Y., Xin X., Xu D., Yan R., Xu L., Shen B., Hou L. (2023). Grassland Ecosystem Progress: A Review and Bibliometric Analysis Based on Research Publication over the Last Three Decades. Agronomy.

[2] Zhao Y., Liu Z., Wu J. (2020). Grassland ecosystem services: A systematic review of research advances and future directions. Landsc. Ecol..

[3] Chen Y., Feng J., Yuan X., Zhu B. (2020). Effects of warming on carbon and nitrogen cycling in alpine grassland ecosystems on the Tibetan Plateau: A meta-analysis. Geoderma.

[4] Bengtsson J., Bullock J.M., Egoh B., Everson C., Everson T., O’connor T., O’farrell P., Smith H.G., Lindborg R. (2019). Grasslands—More important for ecosystem services than you might think. Ecosphere.

[5] Fang J., Yang Y., Ma W., Mohammat A., Shen H. (2010). Ecosystem carbon stocks and their changes in China’s grasslands. Sci. China Life Sci..

[6] Zhang C.H., Guo H.R., Huang H., Ma T.Y., Song W., Chen C.J., Liu X.Y. (2021). Atmospheric nitrogen deposition and its responses to anthropogenic emissions in a global hotspot region. Atmos. Res..

[7] Liu X., Zhang Y., Han W., Tang A., Shen J., Cui Z., Vitousek P., Erisman J.W., Goulding K., Christie P. (2013). Enhanced nitrogen deposition over China. Nature.

[8] Yu G., Jia Y., He N., Zhu J., Chen Z., Wang Q., Piao S., Liu X., He H., Guo X. (2019). Stabilization of atmospheric nitrogen deposition in China over the past decade. Nat. Geosci..

[9] Zhang R., Schellenberg M.P., Tian D., Ma F., Zhang T., Wang H., Wu Q., Bai Y., Han G., Niu S. (2021). Shifting community composition determines the biodiversity–productivity relationship under increasing precipitation and N deposition. J. Veg. Sci..

[10] Verma P., Sagar R. (2020). Responses of diversity, productivity, and stability to the nitrogen input in a tropical grassland. Ecol. Appl..

[11] Lu P., Hao T., Li X., Wang H., Zhai X., Tian Q., Bai W., Stevens C., Zhang W.H. (2021). Ambient nitrogen deposition drives plant-diversity decline by nitrogen accumulation in a closed grassland ecosystem. J. Appl. Ecol..

[12] Liu S., Yang R., Peng X., Hou C., Ma J., Guo J. (2022). Contributions of Plant Litter Decomposition to Soil Nutrients in Ecological Tea Gardens. Agriculture.

[13] Liu Q., Xu H., Yi H. (2021). Impact of Fertilizer on Crop Yield and C:N:P Stoichiometry in Arid and Semi-Arid Soil. Int. J. Environ. Res. Public Health.

[14] Tian D., Niu S. (2015). A global analysis of soil acidification caused by nitrogen addition. Environ. Res. Lett..

[15] Xu C., Xu X., Ju C., Chen H.Y., Wilsey B.J., Luo Y., Fan W. (2021). Long-term, amplified responses of soil organic carbon to nitrogen addition worldwide. Glob. Chang. Biol..

[16] Zhou Z., Wang C., Zheng M., Jiang L., Luo Y. (2017). Patterns and mechanisms of responses by soil microbial communities to nitrogen addition. Soil Biol. Biochem..

[17] Lu X., Mo J., Gilliam F.S., Fang H., Zhu F., Fang Y., Zhang W., Huang J. (2012). Nitrogen Addition Shapes Soil Phosphorus Availability in Two Reforested Tropical Forests in Southern China. Biotropica.

[18] Schleuss P.M., Widdig M., Heintz-Buschart A., Guhr A., Martin S., Kirkman K., Spohn M. (2019). Stoichiometric controls of soil carbon and nitrogen cycling after long-term nitrogen and phosphorus addition in a mesic grassland in South Africa. Soil Biol. Biochem..

[19] Tian J., Dungait J.A., Lu X., Yang Y., Hartley I.P., Zhang W., Mo J., Yu G., Zhou J., Kuzyakov Y. (2019). Long-term nitrogen addition modifies microbial composition and functions for slow carbon cycling and increased sequestration in tropical forest soil. Glob. Chang. Biol..

[20] Chen D., Xing W., Lan Z., Saleem M., Wu Y., Hu S., Bai Y. (2019). Direct and indirect effects of nitrogen enrichment on soil organisms and carbon and nitrogen mineralization in a semi-arid grassland. Funct. Ecol..

[21] Aber J., McDowell W., Nadelhoffer K., Magill A., Berntson G., Kamakea M., McNulty S., Currie W., Rustad L., Fernandez I. (1998). Nitrogen Saturation in Temperate Forest Ecosystems: Hypotheses revisited. BioScience.

[22] Elrys A.S., Wang J., Meng L., Zhu Q., El-Sawy M.M., Chen Z., Tu X., El-Saadony M.T., Zhang Y., Zhang J. (2023). Integrative knowledge-based nitrogen management practices can provide positive effects on ecosystem nitrogen retention. Nat. Food.

[23] Gurmesa G.A., Hobbie E.A., Zhang S., Wang A., Zhu F., Zhu W., Koba K., Yoh M., Wang C., Zhang Q. (2022). Natural ^15^N abundance of ammonium and nitrate in soil profiles: New insights into forest ecosystem nitrogen saturation. Ecosphere.

[24] Gilliam F.S. (2006). Response of the herbaceous layer of forest ecosystems to excess nitrogen deposition. J. Ecol..

[25] Humbert J.Y., Dwyer J.M., Andrey A., Arlettaz R. (2016). Impacts of nitrogen addition on plant biodiversity in mountain grasslands depend on dose, application duration and climate: A systematic review. Glob. Chang. Biol..

[26] Lu M., Yang Y., Luo Y., Fang C., Zhou X., Chen J., Yang X., Li B. (2011). Responses of ecosystem nitrogen cycle to nitrogen addition: A meta-analysis. New Phytol..

[27] Zhang Y., Loreau M., Lü X., He N., Zhang G., Han X. (2016). Nitrogen enrichment weakens ecosystem stability through decreased species asynchrony and population stability in a temperate grassland. Glob. Chang. Biol..

[28] Wang W., Tang J., Zhang N., Wang Y., Xu X., Zhang A. (2023). Spatiotemporal Pattern of Invasive Pedicularis in the Bayinbuluke Land, China, during 2019–2021: An Analysis Based on PlanetScope and Sentinel-2 Data. Remote Sens..

[29] Li K., Liu X., Song L., Gong Y., Lu C., Yue P., Tian C., Zhang F. (2015). Response of alpine grassland to elevated nitrogen deposition and water supply in China. Oecologia.

[30] Bobbink R., Hicks K., Galloway J., Spranger T., Alkemade R., Ashmore M., Bustamante M., Cinderby S., Davidson E., Dentener F. (2010). Global assessment of nitrogen deposition effects on terrestrial plant diversity: A synthesis. Ecol. Appl..

[31] Liang Y.Y., Zhang L.X., Zhou X.L., Fan L.L., Mao J.F., Li Y.M. (2023). Response of soil aggregate structure and nutrient content to simulated nitrogen and phosphorus deposition in alpine grassland of Tianshan Mountain. J. Ecol..

[32] Bao S.D. (2000). Soil Agrochemical Analysis.

[33] Zhang J., Zuo X., Lv P. (2023). Effects of Grazing, Extreme Drought, Extreme Rainfall and Nitrogen Addition on Vegetation Characteristics and Productivity of Semiarid Grassland. Int. J. Environ. Res. Public Health.

[34] Li J., Charles L.S., Yang Z. (2022). Differential Mechanisms Drive Species Loss under Artificial Shade and Fertilization in the Alpine Meadow of the Tibetan Plateau. Front. Plant Sci..

[35] Lu X., Gilliam F.S., Guo J., Hou E., Kuang Y. (2022). Decrease in soil pH has greater effects than increase in above-ground carbon inputs on soil organic carbon in terrestrial ecosystems of China under nitrogen enrichment. J. Appl. Ecol..

[36] Band N., Kadmon R., Mandel M., DeMalach N. (2022). Assessing the roles of nitrogen, biomass, and niche dimensionality as drivers of species loss in grassland communities. Proc. Natl. Acad. Sci. USA.

[37] Bai Y., Wu J., Clark C.M., Naeem S., Pan Q., Huang J., Zhang L., Han X. (2010). Tradeoffs and thresholds in the effects of nitrogen addition on biodiversity and ecosystem functioning: Evidence from inner Mongolia Grasslands. Glob. Chang. Biol..

[38] Van Sundert K., Arfin Khan M.A., Bharath S., Buckley Y.M., Caldeira M.C., Donohue I., Dubbert M., Ebeling A., Eisenhauer N., Eskelinen A. (2021). Fertilized graminoids intensify negative drought effects on grassland productivity. Glob. Chang. Biol..

[39] He K., Huang Y., Qi Y., Sheng Z., Chen H. (2021). Effects of nitrogen addition on vegetation and soil and its linkages to plant diversity and productivity in a semi-arid steppe. Sci. Total Environ..

[40] Li W., Wen S., Hu W., Du G. (2011). Root–shoot competition interactions cause diversity loss after fertilization: A field experiment in an alpine meadow on the Tibetan Plateau. J. Plant Ecol..

[41] DeMalach N., Kadmon R. (2017). Light competition explains diversity decline better than niche dimensionality. Funct. Ecol..

[42] Yang B., Qiao N., Xu X., Ouyang H. (2011). Symbiotic nitrogen fixation by legumes in two Chinese grasslands estimated with the ^15^N dilution technique. Nutr. Cycl. Agroecosystems.

[43] Mao J., Mao Q., Zheng M., Mo J. (2020). Responses of Foliar Nutrient Status and Stoichiometry to Nitrogen Addition in Different Ecosystems: A Meta-analysis. J. Geophys. Res. Biogeosciences.

[44] Sterner R.W., Elser J.J. (2003). Ecological Stoichiometry: The Biology of Elements from Molecules to the Biosphere.

[45] Pang Y., Tian J., Wang D. (2021). Response of multi-ecological component stoichiometry and tree nutrient resorption to medium-term whole-tree harvesting in secondary forests in the Qinling Mountains, China. For. Ecol. Manag..

[46] Zhang Y., Mai H., Qiu Q., Zhu Y., Long J., Chen S., Chen Y. (2023). The Responses of C, N, P and Stoichiometric Ratios to Biochar and Vermicompost Additions Differ from Alfalfa and a Mine Soil. Agriculture.

[47] Long M., Wu H.H., Smith M.D., La Pierre K.J., Lü X.T., Zhang H.Y., Han X.G., Yu Q. (2016). Nitrogen deposition promotes phosphorus uptake of plants in a semi-arid temperate grassland. Plant Soil.

[48] Koerselman W., Meuleman A.F.M. (1996). The Vegetation N:P Ratio: A New Tool to Detect the Nature of Nutrient Limitation. J. Appl. Ecol..

[49] Güsewell S. (2004). N : P ratios in terrestrial plants: Variation and functional significance. New Phytol..

[50] Xie Y., Qiu K., Xu D., Shi X., Qi T., Pott R. (2015). Spatial heterogeneity of soil and vegetation characteristics and soil-vegetation relationships along an ecotone in Southern Mu Us Sandy Land, China. J. Soils Sediments.

[51] Deekshitha D.K.D., Rao C.S., Subbaiah P.V., Luther M.M., Rao V.S. (2021). Direct and Residual Effect of Integrated Nitrogen Management on Physico-Chemical Properties of Soil. Int. J. Environ. Clim. Chang..

[52] Wang Y., Wang J., Guo D., Zhang H., Che Y., Li Y., Tian B., Wang Z., Sun G., Zhang H. (2021). Physiological and comparative transcriptome analysis of leaf response and physiological adaption to saline alkali stress across pH values in alfalfa (*Medicago sativa*). Plant Physiol. Biochem..

[53] Gao Y., Sun S., Xing F., Mu X., Bai Y. (2019). Nitrogen addition interacted with salinity-alkalinity to modify plant diversity, microbial PLFAs and soil coupled elements: A 5-year experiment. Appl. Soil Ecol..

[54] Han J., Shi J., Zeng L., Xu J., Wu L. (2015). Effects of nitrogen fertilization on the acidity and salinity of greenhouse soils. Environ. Sci. Pollut. Res..

[55] Ma L.N., Lü X.T., Liu Y., Guo J.X., Zhang N.Y., Yang J.Q., Wang R.Z. (2011). The Effects of Warming and Nitrogen Addition on Soil Nitrogen Cycling in a Temperate Grassland, Northeastern China. PLoS ONE.

[56] Alam S.M., Naqvi S.S.M., Ansari R. (1999). Impact of soil pH on nutrient uptake by crop plants. Handbook of Plant and Crop Stress.

[57] Zhang R., Nie L., Huang M., Yang H., Shi C., Wei Y., Song L., Zhu J., Bo H., Wang J. (2022). Effects of Irrigation and Nitrogen Application on Soil Nutrients in Triploid Populus tomentosa Stands. Forests.

[58] Chen J., Xiao W., Zheng C., Zhu B. (2020). Nitrogen addition has contrasting effects on particulate and mineral-associated soil organic carbon in a subtropical forest. Soil Biol. Biochem..

[59] Fathi A. (2022). Role of nitrogen (N) in plant growth, photosynthesis pigments, and N use efficiency: A review. Agrisost.

[60] Gao W., Yang H., Kou L., Li S. (2015). Effects of nitrogen deposition and fertilization on N transformations in forest soils: A review. J. Soils Sediments.

[61] Devau N., Le Cadre E., Hinsinger P., Jaillard B., Gérard F. (2009). Soil pH controls the environmental availability of phosphorus: Experimental and mechanistic modelling approaches. Appl. Geochem..

[62] Margalef O., Sardans J., Fernández-Martínez M., Molowny-Horas R., Janssens I.A., Ciais P., Goll D., Richter A., Obersteiner M., Asensio D. (2017). Global patterns of phosphatase activity in natural soils. Sci. Rep..

[63] Deng Q., Hui D., Dennis S., Reddy K.C. (2017). Responses of terrestrial ecosystem phosphorus cycling to nitrogen addition: A meta-analysis. Glob. Ecol. Biogeogr..

[64] Huang J.Y., Lai R.S., Yu H.L., Chen W.M. (2013). Effects of N addition on the ecological stoichiometric characteristics of plant and soil C:N:P in Ningxia desert grassland. J. Ecol..

[65] Sun X., Yu K., Shugart H.H., Wang G. (2016). Species richness loss after nutrient addition as affected by N:C ratios and phytohormone GA3 contents in an alpine meadow community. J. Plant Ecol..

[66] Wang N., Xu S.S., Jia X., Gao J., Zhang W.P., Qiu Y.P., Wang G.X. (2013). Variations in foliar stable carbon isotopes among functional groups and along environmental gradients in China—A meta-analysis. Plant Biol..

[67] Fayiah M., Dong S., Li Y., Xu Y., Gao X., Li S., Shen H., Xiao J., Yang Y., Wessell K. (2019). The relationships between plant diversity, plant cover, plant biomass and soil fertility vary with grassland type on Qinghai-Tibetan Plateau. Agric. Ecosyst. Environ..

